# Clinical study replicability and the pursuit of excellence

**DOI:** 10.1186/s13054-015-1019-1

**Published:** 2015-08-24

**Authors:** Michael J. Lanspa, Eliotte L. Hirshberg, Russell R. Miller, Alan H. Morris

**Affiliations:** Division of Pulmonary and Critical Care Medicine, Intermountain Medical Center, 5121 S. Cottonwood Street, Murray, UT 84107 USA; Division of Respiratory, Critical Care and Occupational Pulmonary Medicine, University of Utah, 26 North 1900 East, Salt Lake City, UT 84132 USA; Division of Pediatric Critical Care, Department of Pediatrics, University of Utah, 295 Chipeta Way, Salt Lake City, UT 84108 USA

## Abstract

Comparisons of processes of care are common in critical care research. Often, these processes are neither explicit nor replicable and this can result in seemingly irreconcilable results. Here, we briefly review the article by Taniguchi and colleagues, who studied liberation from mechanical ventilation by using either a computerized weaning protocol or one driven by respiratory therapists. We discuss the implications of explicit protocols increasing replicability in clinical research.

In a recent article published in *Critical Care*, Taniguchi and colleagues [1] compared weaning time in 70 critically ill patients by using either a respiratory physiotherapist-guided protocol or a proprietary, adaptive, closed-loop protocol: SmartCare™ (Dräger, Lübeck, Germany). The authors found that the two protocols resulted in similar times of mechanical ventilation support but that the physiotherapist protocol had a shorter weaning time.

Both study protocols are sophisticated. The physiotherapist-guided protocol accounted for gas exchange and airway pressures as well as hemodynamic stability, arrhythmia, and vasopressor use. Although both protocols are detailed and explicit, this study is not immune to the problem prevalent in so many studies of ventilator weaning: It is exceedingly difficult to account for all of the clinical issues related to ventilation, such as clinical complications, physician workload, and patient sedation. Weaning time may be less a result of a specific protocol than of sedation practices [2, 3]. Therefore, many decisions about ventilator weaning and spontaneous breathing trials are based on the practice patterns and biases of individual clinicians [4].

It appears that, although subjects in the study by Taniguchi and colleagues were supported with similar times of mechanical ventilation, the authors terminated weaning at different ventilator settings. The physiotherapist group terminated weaning at pressures of 8 cm H_2_O, whereas the SmartCare™ group terminated weaning at 5 cm H_2_O. Terminating weaning at the higher pressure (8 cm H_2_O) in the physiotherapist group may have been the major determinant of the shorter weaning time. The authors acknowledge that the SmartCare™ group experienced a greater number of clinical complications *unrelated to* the ventilation support mode. These complications may have altered the results in favor of the physiotherapist group.

Although the Hawthorne effect is often overstated, it may be present. Non-blinded studies that rely on usual care may also be susceptible to the influence of the research environment [5]. As an example, adherence to low-stretch ventilation for acute respiratory distress syndrome was much higher when it was part of a study than in the usual care after conclusion of the study [6]. It is possible that the care administered during the study by Taniguchi and colleagues was different from their local usual care. However, the key finding (good physiotherapists, working at their best, are superior to a sophisticated automated protocol) is not dependent on whether the study care reflected usual care.

Taniguchi and colleagues demonstrated that weaning by Albert Einstein Israelite Hospital (São Paulo, Brazil) respiratory physiotherapists is equivalent to, or better than, weaning by the SmartCare™ system. In contrast, several other institutions found the SmartCare™ system to be superior to their local usual care [7]. Although much of the critical care literature compares processes of care, few studies compare adequately detailed processes; many use some version of “usual care” or a protocol that requires clinician judgment and allows considerable intra- and inter-clinician variation. Variations in processes of care can result in seemingly irreconcilable results. Considering previous studies, one might have incorrectly concluded that SmartCare™ was better than respiratory physiotherapist-driven weaning. The reason that Taniguchi and colleagues report a contrary result is likely because the Albert Einstein Israelite Hospital weaning protocol is different than, and presumably superior to, other “usual care” protocols.

The excellent outcomes of ventilation duration, weaning time, and successful extubation in the respiratory physiotherapist group serve as a testament to the quality of the care provided by physicians and respiratory physiotherapists at Albert Einstein Israelite Hospital. Taniguchi and colleagues speculate that the shorter spontaneous breathing trial and extubation times may be attributable to the superior ability of these clinicians to tailor therapy to individual patient needs. How one might generalize this study’s findings to one’s own practice, when the physiotherapy protocol relies in part on clinician judgment, is unclear. Exporting this protocol to an institution with different practices may yield different results. Not every respiratory physiotherapist will be comparable to those who performed this study. The unanswered question is whether other institutions, adopting the Albert Einstein protocol, would achieve outcomes comparable to those reported by Taniguchi and colleagues. The more detailed the protocol rules, the more likely the protocol is replicable. The Albert Einstein protocol is an excellent step in that direction, as it is detailed and offers far less opportunity for inter-clinician variability than is present in several other studies of SmartCare™ versus undirected weaning strategies [7]. However, no open-loop protocol (requiring clinician judgment) is perfectly replicable. Perhaps more enticing is the possibility for future iterations of a closed-loop computer protocol with evidence-based, detailed rules that capture the decision-making processes of our best clinicians.

## Conclusions

As more critical care investigations study processes of care and report seemingly contradictory results, we are obliged to consider the replicability of the study method. Rigorous studies require detailed protocols that avoid unnecessary variation. The impressive results of the Albert Einstein clinicians underscore the need to dissect and replicate their decision-making processes. The fewer the opportunities for intra- and inter-clinician variation, the less dependent the results will be on the talents of one group of clinicians.
